# Glycoprotein Hyposialylation Gives Rise to a Nephrotic-Like Syndrome That Is Prevented by Sialic Acid Administration in GNE V572L Point-Mutant Mice

**DOI:** 10.1371/journal.pone.0029873

**Published:** 2012-01-13

**Authors:** Mitutoshi Ito, Kazushi Sugihara, Tomoya Asaka, Tadashi Toyama, Toru Yoshihara, Kengo Furuichi, Takashi Wada, Masahide Asano

**Affiliations:** 1 Division of Transgenic Animal Science, Advanced Science Research Center, Kanazawa University, Kanazawa, Japan; 2 Nanao National Hospital, Nanao, Japan; 3 Research Center for Child Mental Development, Kanazawa University, Kanazawa, Japan; 4 Department of Laboratory Medicine, Kanazawa University Graduate School of Medical Science, Kanazawa, Japan; Kanazawa University, Japan

## Abstract

Mutations in the key enzyme of sialic acid biosynthesis, UDP-*N*-acetylglucosamine 2-epimerase/*N*-acetyl-mannosamine kinase, result in distal myopathy with rimmed vacuoles (DMRV)/hereditary inclusion body myopathy (HIBM) in humans. Sialic acid is an acidic monosaccharide that modifies non-reducing terminal carbohydrate chains on glycoproteins and glycolipids, and it plays an important role in cellular adhesions and interactions. In this study, we generated mice with a V572L point mutation in the GNE kinase domain. Unexpectedly, these mutant mice had no apparent myopathies or motor dysfunctions. However, they had a short lifespan and exhibited renal impairment with massive albuminuria. Histological analysis showed enlarged glomeruli with mesangial matrix deposition, leading to glomerulosclerosis and abnormal podocyte foot process morphologies in the kidneys. Glycan analysis using several lectins revealed glomerular epithelial cell hyposialylation, particularly the hyposialylation of podocalyxin, which is one of important molecules for the glomerular filtration barrier. Administering Neu5Ac to the mutant mice from embryonic stages significantly suppressed the albuminuria and renal pathology, and partially recovered the glomerular glycoprotein sialylation. These findings suggest that the nephrotic-like syndrome observed in these mutant mice resulted from impaired glomerular filtration due to the hyposialylation of podocyte glycoproteins, including podocalyxin. Furthermore, it was possible to prevent the nephrotic-like disease in these mice by beginning Neu5Ac treatment during gestation.

## Introduction

**Figure 1:**
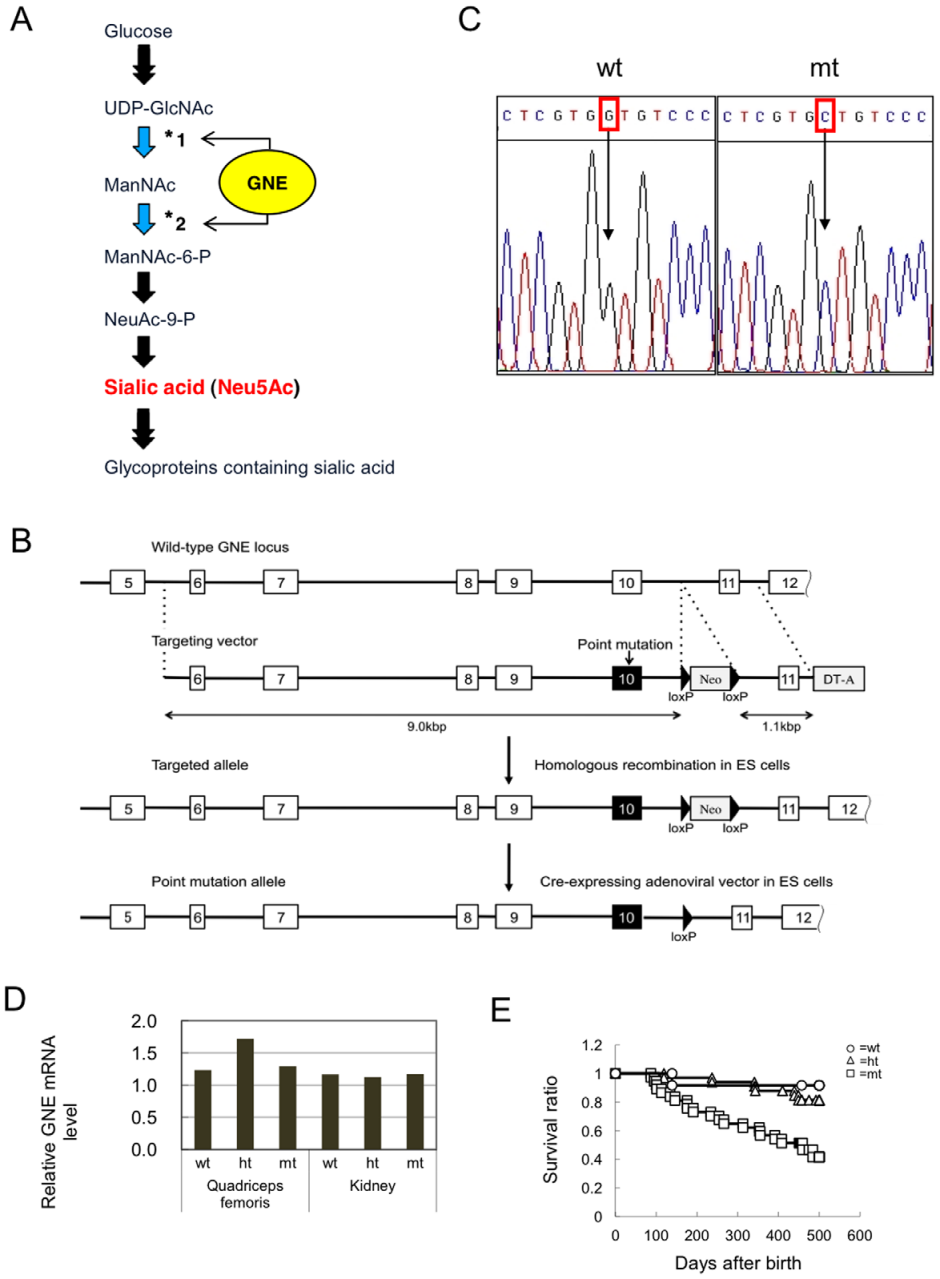
The sialic acid biosynthesis pathway and the generation of GNE V572L point-mutant mice. (**A**) The sialic acid biosynthesis pathway. GNE has dual-function enzymatic activity, UDP-*N*-acetylglucosamine-2-epimerase (*1) and *N*-acetylmannosamine kinase (*2), in the cytosol. (**B**) Targeting strategy to create a point mutation in exon 10 of the mouse *GNE* gene: to make the targeted allele, the wild-type GNE locus was replaced with a targeting vector, which contained a point mutation in exon 10, a neo cassette and a DT-A cassette, by homologous recombination. The neo cassette, flanked by two loxP sites, was deleted by a Cre-expressing adenoviral vector to make the point mutation allele. Neo, neomycin-resistance gene; DT-A, diphtheria toxin A fragment; triangles, loxP sites; open boxes with numbers, exons; closed boxes, exon 10 containing the point mutation. (**C**) A base exchange from G (wild-type; wt) to C (mutant; mt) at the 1714 site was confirmed by DNA sequencing. (**D**) Quantitative RT-PCR analysis of the *GNE* mRNA level normalized to the *GAPDH* mRNA level in the quadriceps femoris and kidney of the mutant (mt), heterozygous (ht), and wild-type (wt) mice. (**E**) Survival ratio of mt (squares, n = 37), ht (triangles, n = 33), and wt mice (circles, n = 12), analyzed by Kaplan Meier methods.

UDP-*N*-acetylglucosamine 2-epimerase/*N*-acetyl-mannosamine kinase is a dual-function enzyme that catalyzes the rate-limiting step in sialic acid biosynthesis (Figure 1A) [1]. Mice with a null mutation in the *GNE* gene are embryonic lethal, indicating that GNE is essential for early embryonic development [2]. Human *GNE* mutations result in an adult-onset, progressive, autosomal recessive muscular disorder, distal myopathy with rimmed vacuoles (DMRV)/hereditary inclusion body myopathy (HIBM) [3]–[5]. Among the various *GNE* mutations, one *GNE* founder mutation (V572L) has been reported in Japanese families affected by DMRV [3].

Sialic acid is an acidic monosaccharide known to modify non-reducing terminal carbohydrates on glycoproteins and glycolipids, where it functions in cellular adhesions and interactions in the nervous and immune systems [6]–[8]. In renal functions, sialic acid residues are important in glomerular filtration, and their deficiency is implicated in proteinuria [9]–[13]. It has been reported that glomerular podocyte and podocyte foot process morphologies are maintained by the anionic charge of sialic acid residues on podocyte glycoproteins and glycolipids [12], [14], and that a barrier to protein permeability is controlled by functional endothelial glycocalyx in glomeruli [15], [16]. The glomerular filtration barrier, which consists of podocytes, the glomerular basement membrane (GBM), and fenestrated endothelial cells, prevents the leakage of albumin and other proteins from the blood stream by size- and charge-dependent filtration [15], [17]. A lack of sialic acid residues on renal glycoproteins and glycolipids neutralizes their negative charge, disrupting the podocyte structure and resulting in massive proteinuria and podocytopathy [9], [13], [18], [19]. For instance, it was previously shown that loss of podocyte foot processes was induced by the injection of puromycin aminonucleoside to neutralize the glomerular negative charge in normal rats [13], [19]. However, it is still not clear whether the development of proteinuria is caused by the defects of sialic acid residues on podocyte glycoproteins and glycolipids.

To develop an animal DMRV model and clarify the role of sialic acid residues in the development of DMRV or other diseases, we generated mice with a kinase-domain point mutation (V572L) in GNE. Surprisingly, there were no apparent myopathic features or motor dysfunctions in the GNE V572L point-mutant homozygous mice (mt-mice). However, the mt-mice had a short lifespan, massive proteinuria after birth, and abnormal kidney morphology.

Other than *GNE-*null mice, two *GNE* mutant mice have been reported previously. Transgenic mice expressing a human D176V point-mutant *GNE* gene in a mouse *GNE* knockout background develop myopathic disorders similar to DMRV [20], which are rescued by administering sialic acid metabolites [21]. However, no renal features have been described in these mice. On the other hand, another knock-in mouse carrying a GNE M712T point mutation cannot survive beyond 3 days due to severe glomerular hematuria, proteinuria, and podocytopathy [18]. Administering *N*-acetyl-mannosamine (ManNAc) partially suppresses these renal disorders and slightly prolongs their lifespan. However, these mutant mice do not display any myopathic features [18]. It is not clear why these two mice develop such different phenotypes.

Here, we present a third *GNE* mutant mouse, carrying a V572L mutation. This mouse shows renal but not myopathic features, much like the GNE M712T mutant [18]. However, our GNE V572L mutant mice have a much longer lifespan than the GNE M712T mutant. In this study, we examined the effect of hyposialylation caused by the GNE mutation on the renal disorders of the mt-mice, and attempted to suppress the renal disorders by administering 5-*N*-acetylneuraminic acid (Neu5Ac), a major sialic acid. We also evaluated the usefulness of the mt-mouse as an animal model of renal disease with proteinuria, such as congenital nephrotic syndrome (CNS) [17], [22], and the potential of administering Neu5Ac as a therapeutic strategy to prevent renal disorders resulting from hyposialylation.

## Materials and Methods

### Generation of GNE V572L point-mutant mice

Mice containing a V572L point mutation in the *GNE* gene were generated using the ES-cell gene-targeting method. A targeting vector was constructed as follows (Figure 1B). The left arm (9.0 kb), containing exons 6 to 10, and the right arm (1.1 kb), containing exon 11, were amplified by PCR using a BAC clone (male CJ7/129Sv, Research Genetics, Huntsville, AL, USA) containing the mouse *GNE* gene as a template. DNA sequencing verified that there was no PCR error, at least in these exons. Primers used for amplifying exons 6, 7, 8, 9, 10 and 11 are listed in Table S1. A point mutation (G to C at the 1714 site) was created in exon 10 in the left arm using the Gene-editor in vitro Mutagenesis system (Promega Co, Madison, WI) according to the manufacturer's protocol, resulting in a change of Valine (GTG) to Leucine (CTG) at the amino acid 572 site in the GNE kinase domain. A *neo* cassette [23] flanked by two loxP sites was inserted between the left and right arms for positive selection, and a *DT-A* cassette [24] was ligated at the end of the right arm for negative selection. The resulting targeting vector is shown in Figure 1B.

The targeting vector was introduced into E14-1 ES cells (129/Ola strain) [25] by electroporation, and G418-resistant colonies were picked up as described previously [26]. We were only able to obtain one PCR-positive clone out of 670 colonies screened. Primers (GNE screening) used for PCR screening are listed in Table S1. DNA sequencing verified a point mutation in exon 10 of this clone using primers (GNE sequence) listed in Table S1. The homologous recombinant clone was infected with an adenoviral vector expressing the *Cre* gene [27] to delete the *neo* cassette in the genome, as shown in Figure 1B. Chimeric mice generated by the aggregation method [28] were mated with C57BL/6 mice to confirm germ-line transmission. The mt-mice on a mixed 129/Ola and C57BL/6 background, obtained by crossing heterozygous point-mutant mice (ht-mice), were used for experiments. Sex- and age-matched wild-type mice (wt-mice) and ht-mice were used as controls because the ht-mice did not show any renal disorder indistinguishable from wt-mice.

All mice were housed under specific pathogen-free conditions at the Institute for Experimental Animals of Kanazawa University. Animal experiments were conducted in strict accordance with the recommendations in the Fundamental Guidelines for Proper Conduct of Animal Experiments and Related Activities in Academic Research Institutions under the jurisdiction of the Ministry of Education, Culture, Sports, Science and Technology of Japan. The protocol was approved by the Committee on Animal Experimentation of Kanazawa University (Permit Number: AP-111959). All efforts were made to minimize suffering. All experiments were conducted according to the safety guidelines for gene manipulation experiments at Kanazawa University.

### Genotyping by DNA sequencing and PCR

Mice were genotyped by the direct DNA sequencing of PCR products targeting the *GNE* gene exon 10, using genomic DNA taken from the mouse tail (Figure 1C, Table S1). Primers used for PCR are listed in Table S1. Alternatively, PCR genotyping was performed using allele-specific locked nucleic acid (LNA™)-containing primers (Table S1) [29]. The PCR product of the wild-type allele (153 bp) of the *GNE* gene was amplified using primers 1 and 2, while the PCR product of the mutant allele (153 bp) was amplified using primers 1 and 3 (Figure S1A). PCR conditions were as follows: 98°C for 3 min, 20 cycles of 97°C for 20 s, 68.5°C for 30 s, and 72°C for 25 s, followed by 25 cycles of 96°C for 20 s, 52°C for 20 s, and 72°C for 15 s.

### Rota-rod test

To investigate limb motor functions, we used the accelerating rota-rod paradigm [30]. Mice were tested in 3 trials per day for 3 consecutive days with a 300-s accelerating program (from 5 to 40 rpm), and the latency of the animal to fall from the rod was recorded.

### Clinical chemistry test

Mice were placed individually in metabolic cages (Shinano Factory Co., Tokyo, Japan) for 24 hours with free access to food and water. Urine samples and heart serum samples were collected. Clinical chemistry tests of these samples, performed at the Nagahama Life Science Laboratory, measured the urine albumin, urine creatinine, serum albumin, and serum cystatin C. To analyze proteinuria in postnatal mice, urine samples (8 µl) were applied directly to SDS polyacrylamide gel electrophoresis (SDS-PAGE), and albumin was detected by Coomassie Brilliant Blue (CBB) staining.

### Antibodies and lectins

The following primary antibodies and lectins were used for experiments: goat anti-mouse podocalyxin (R&D Systems, Inc., Minneapolis, MN), goat anti-mouse podoplanin (R&D Systems, Inc.), rabbit anti-mouse nephrin (Abcam, Tokyo, Japan), rabbit anti-mouse ezrin (Abcam), goat anti-mouse NHERF2 (Santa Cruz, Santa Cruz, CA), rabbit anti-mouse podocin (Santa Cruz), rabbit anti-mouse NEPH1 (Abcam), biotin-conjugated *Peanut agglutinin* (PNA) lectin (Seikagaku Biobusiness Co., Tokyo, Japan), biotin-conjugated *Ricinus communis Agglutinin* (RCA-I) lectin (Seikagaku Biobusiness Co.), *Dolichos biflorus agglutinin* (DBA) lectin (Vector Laboratories, Inc., Burlingame, CA), fluorescein isothiocyanate (FITC)-conjugated *Lotus tetragonolobus* (LTA) lectin (Vector Laboratories, Inc.), FITC-conjugated *Lycopersicon Esculentum* (LEL) lectin (Vector Laboratories, Inc.), and Lectin Kit I, Fluorescein Labeled (Vector Laboratories, Inc, FLK-2100). PNA, RCA-I, DBA, LTA, and LEL recognized the following glycan structures: Galβ1-3GalNAc, Galβ1-4GlcAc, GalNAcα1-3GalNAc, L-Fucose, and GlcNAc oligomers, respectively.

The following secondary antibodies were used for experiments: Alexa Fluor 546-conjugated donkey anti-goat IgG (Life Technologies Japan, Ltd.,Tokyo, Japan), Alexa Fluor 488-conjugated goat anti-rabbit IgG (Molecular Probes, Eugene, OR), biotinylated goat anti-rabbit IgG (Vector Laboratories, Inc.), and biotinylated rabbit anti-goat IgG (Vector Laboratories, Inc.).

### Histological analysis

Mouse kidneys were fixed in 10% neutral buffered formalin or 4% paraformaldehyde (PFA), respectively, in phosphate-buffered saline (PBS) (pH 7.4), then dehydrated and embedded in paraffin according to standard procedures. Tissue was sectioned (5 µm), deparaffinized, rehydrated, and stained with Periodic acid-Schiff (PAS) or Van-Gieson staining (a mixture of picric acid and acid fuchsin), each using standard methods. Fresh-frozen sections (10 µm) of mouse skeletal muscles were stained with modified Gomori trichrome staining method. For immunofluorescent staining, sections were deparaffinized and blocked with SuperBlock blocking buffer (Pierce Biotechnology, Inc., Rockford, IL). They were incubated with anti-PC antibody (1/200 dilution) at 4°C overnight, incubated with Alexa Fluor 546-conjugated or Alexa Fluor 488-conjugated secondary antibodies (1/200 dilution) at room temperature (r.t.) for 2 hours, and mounted in ProLong Gold antifade reagent (Molecular Probes). Nuclei were stained with 4′, 6-diamidine-2-phenylindole (DAPI). Stained sections were observed under a fluorescence microscope (Olympus IX 71; Olympus, Corp., Tokyo, Japan) and confocal laser-scanning microscope (LMS510 META; Zeiss, Inc., Thornwood, NY).

For lectin staining, sections were deparaffinized and blocked with 0.1% Tween-20 in Tris-buffered saline (TBS) (pH 7.4), incubated with FITC-conjugated lectins (PNA, RCA-I, LTA, and DBA diluted 1/200) at r.t. for 2 hours, then mounted and observed.

### Electron microscopy

Mouse kidneys were fixed with glutaraldehyde and osmium tetroxide, embedded in Epon 812 (Oken Shoji Co., Tokyo, Japan), and sliced into 0.1 µm sections. Sections were double-stained with uranyl acetate and lead citrate, and examined under an electron microscope (JEM-1210; JEOL Ltd., Tokyo, Japan) [31], [32].

### Western and lectin blotting

Frozen mouse kidneys were homogenized and dissolved in RIPA buffer, consisting of 50 mM Tris-HCl (pH 7.5), 150 mM NaCl, 1 mM EDTA, 0.5% NP-40, 0.5% sodium deoxycholate, 1.0% TritonX-100, and 0.1% SDS. Tissue lysates were centrifuged at 12,000× g at 4°C for 15 minutes to remove insoluble debris. The supernatants were mixed with Laemmli's sample buffer, consisting of 3% SDS, 5% glycerol, 1.67 mM Tris-HCl (pH 7.5), 0.05% bromophenol blue, and 10% 2-mercapto-ethanol, then boiled at 100°C for 3 minutes. The lysate protein concentration was measured using a BCA™ Protein Assay Kit (Pierce Biotechnology, Inc.) according to the manufacturer's protocol.

For Western blotting, proteins were separated by 12% or 8% SDS-PAGE using the Laemmli's buffer system, then transferred to PVDF membranes (Millipore Corp., Bedford, MA). After blocking with Block Ace (Yukijirushi Co, Ltd., Tokyo, Japan), the membranes were incubated overnight with primary antibodies (1/1000 or 1/500 dilution) at 4°C. After the membranes were treated with secondary antibody (1/2000 dilution) at r.t. for 30 minutes, the protein bands were detected with a Vectastain *Elite* ABC standard kit (Vector Laboratories, Inc.) and a Metal Enhanced DAB Substrate Kit (Thermo Scientific Inc., Rockford, IL) according to the manufacturers' protocols.

For lectin blotting, in brief, proteins were separated and transferred to PVDF membranes, as for Western blotting. After blocking with 0.05% Tween-20 in TBS, the membranes were incubated overnight with biotin-conjugated lectins at 4°C, and protein bands were detected as for Western blotting.

For neuraminidase treatment, 1 mU/µg neuraminidase from *Arthrobacter ureafaciens* (Nacalai Tesque, Inc., Kyoto, Japan) was added to the soluble proteins in the RIPA buffer and incubated at 37°C for 30 minutes. The desialylated proteins were mixed with Laemmli's sample buffer and boiled at 100°C for 3 minutes, and the lysates were used for Western blotting.

### Immunoprecipitation

For immunoprecipitation, Protein G Sepharose 4 Fast Flow (GE Healthcare, Upsala, Sweden) was used according to the manufacturer's protocol. In brief, soluble kidney lysates prepared as the above Western blotting method were incubated with anti-PC antibody at 4°C for 1 hour with rotating, and then incubated with 50 µl of Protein G Sepharose at 4°C for 1 hour with rotating. Precipitated proteins were recovered by spin-down, washed, and then dissolved in Laemmli's sample buffer. The precipitated proteins were analyzed by Western and lectin blotting as described above.

### Neu5Ac rescue experiments

Neu5Ac was administered as follows (Figure S2): briefly, pregnant ht-mice were left untreated or were treated with Neu5Ac (Nacalai Tesque, Inc.) (1 g/kg/day) in drinking water, from the time of mating through the nursing period. After weaning, pups were left untreated or were treated with Neu5Ac (0.2 g/kg/day) in the drinking water until 2 months of age; these Neu5Ac doses were selected based on other studies. [18], [21]. The treated mice were sacrificed at 2 months old and analyzed by histological and biochemical methods.

### Quantitative RT-PCR

Total RNA was extracted from mouse kidneys by the guanidine isothiocyanate method [33]. Complementary DNA (cDNA) was synthesized by a PrimeScript RT reagent kit (Takara Bio, Inc., Shiga, Japan), and real-time PCR amplification was performed using a Thermal Cycler Dice (Takara Bio, Inc.) with SYBR Premix Ex Taq II (Takara Bio, Inc.). The primer sequences are listed in Table S1. The PCR conditions were 94°C for 5 minutes, followed by 40 cycles of 94°C for 15 seconds, 60°C for 30 seconds, and a dissociation protocol. The mRNA copy numbers were calculated and normalized to the *GAPDH* mRNA levels.

### Statistics

Statistical evaluation was performed using the Mann-Whitney U-test for clinical chemistry tests or Student's *t*-test for quantitative RT-PCR between the mt-mice and control mice. Two-way ANOVA was used for rescue experiments to confirm the effect of compound treatment and genotypes. A *P*-value<0.05 was considered statistically significant.

## Results

### Generation of GNE V572L point-mutant mice (mt-mice)

We generated mt-mice by the gene targeting method described in Materials and Methods (Figure 1B). A point mutation (G to C) at the 1714 site, resulting in a V572L mutation, was confirmed by DNA sequencing (Figure 1C). *GNE* mRNA levels in the quadriceps femoris and kidney were comparable among the three genotypes—homozygous mutant (mt), heterozygous (ht), and wild-type—in spite of the point mutation (Figure 1D).

The mt-mice were born according to Mendelian inheritance and grew normally with a normal appearance. However, they began to die at around 100 days after birth, and about half of them died by 500 days (Figure 1E). Their kidneys appeared abnormal, being pale and irregularly shaped, but gross examination of various organs revealed no other abnormalities in these mice.

### Severe albuminuria and high serum cystatin C levels in the mt-mice

**Figure 2:**
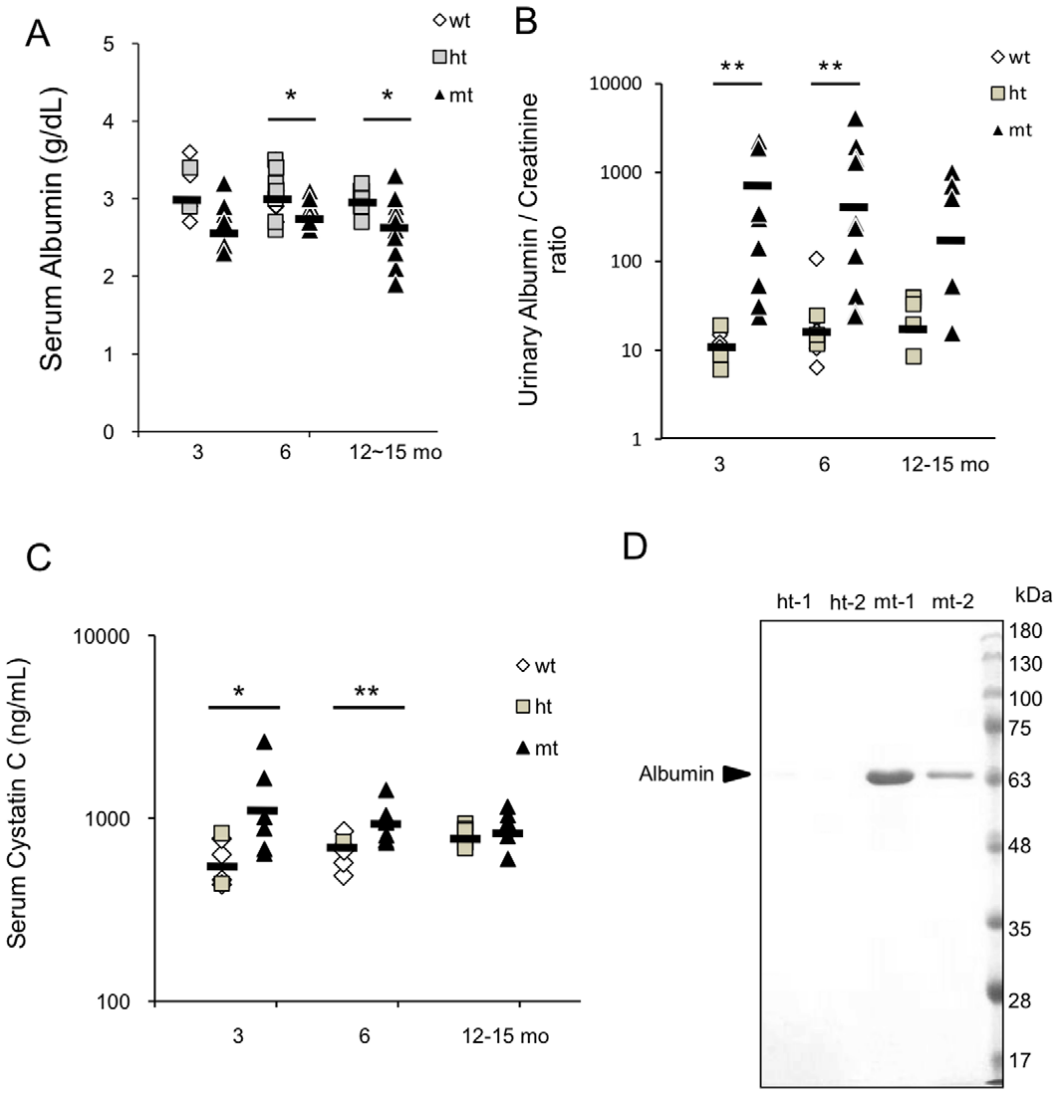
Clinical chemistry tests. (**A**) Serum albumin in wt (open diamonds), ht (gray squares), and mt (black triangles) mice at 3 months (n = 4 wt, 2 ht, 6 mt), 6 months (n = 9 wt, 3 ht, 9 mt), and 12–15 months (n = 1 wt, 11 ht, 12 mt). Data of the wt and ht mice are presented in the same column. (**B**) Urinary albumin/creatinine ratio in wt (open diamonds), ht (gray squares), and mt (black triangles) mice at 3 months (n = 8 wt, 3 ht, 9 mt), 6 months (n = 4 wt, 5 ht, 11 mt), and 12–15 months (n = 5 ht, 5 mt). (**C**) Serum cystatin C at 3 months (n = 4 wt, 2 ht, 6 mt), 6 months (n = 6 wt, 1 ht, 6 mt), and 12–15 months (n = 1 wt, 5 ht, 6 mt). (**D**) SDS-PAGE analysis of urinary proteins in ht and mt mice at 10 days after birth; 8 µl of urine was separated by SDS-PAGE and stained by CBB. Arrowhead: albumin band (60 kDa). Results are shown as means (bars) with individual data points (A, B, C). *P<0.05, **P<0.01 (Mann-Whitney U-test).

We carried out urinary and serologic tests in the mice of various ages. The urinary albumin/creatinine ratio was markedly higher in the mt-mice than in control (wt and ht) mice at 3 and 6 months of age, while the serum albumin levels in the mt-mice were significantly lower than in control mice (Figure 2A, B), suggesting albumin leakage from the blood stream. Moreover, the serum cystatin C levels, which indicate the glomerular filtration rate (GFR) in humans, were also significantly higher in the mt-mice than in control mice (Figure 2C). At 10 days of age, albuminuria was already detectable by SDS-PAGE and CBB staining (Figure 2D). Differences in the urinary albumin/creatinine ratio and serum cystatin C level between mt and ht mice were not significant at 12–15 months old, probably because the severely affected mt-mice had already died, and only the less-affected mt-mice were still alive beyond 1 year.

### Histological analysis of kidneys in the mt-mice

**Figure 3:**
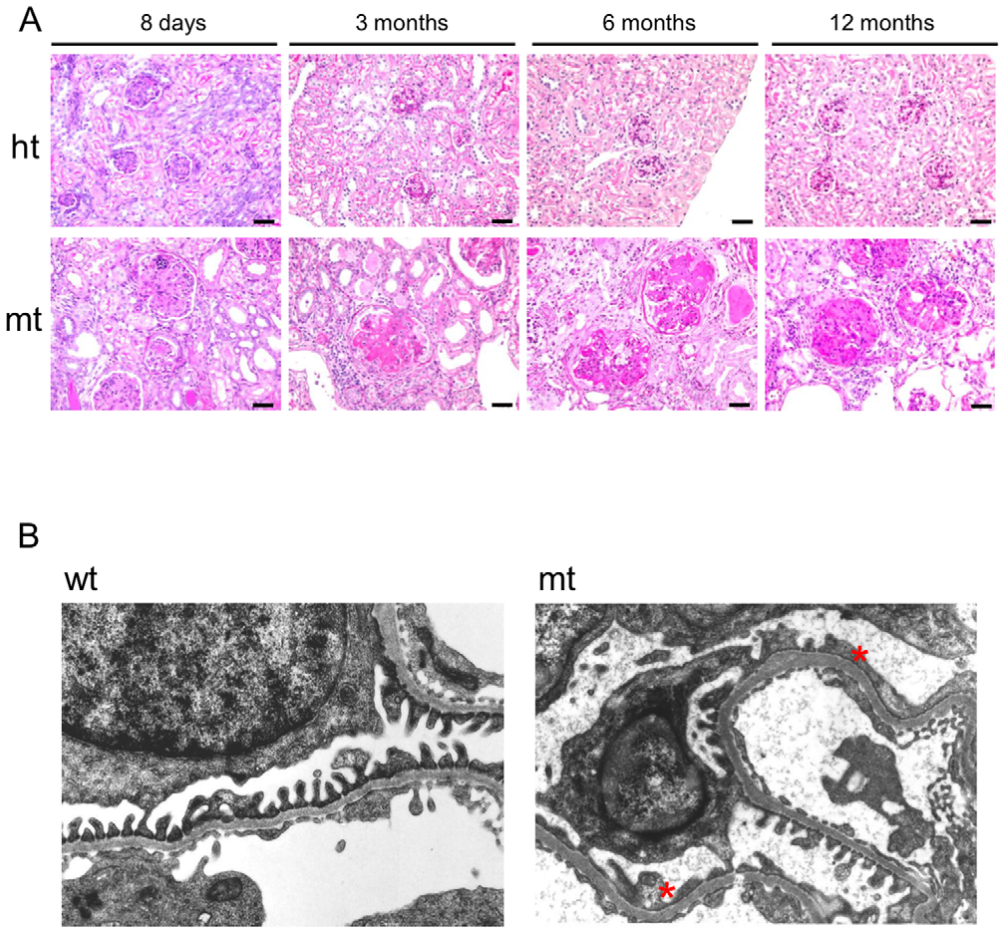
Histological kidney analysis. (A) PAS-stained kidney sections at 8 days, 3 months, 6 months and 12 months of age in ht (upper panels) and mt (lower panels) mice. Scale bars: 20 µm. (B) Electron microscopy of the glomeruli of wt (left panel) and mt mice (right panel) at 4 months old. Asterisks indicate flattened and fused podocyte foot processes.

We examined PAS-stained renal sections of the mt-mice at 8 days, 3 months, 6 months and 12 months of age. At 8 days after birth, cast formation in the renal tubules consistent with albuminuria at the early postnatal stage was observed in the mt-mice. Glomeruli were enlarged in the mt-mice compared to ht-mice (Figure 3A, the first panel from the left). Glomerular lesions with mesangial matrix deposits, including glomerulosclerosis and enlarged Bowman's spaces as well as urinary tubule dilatation with cast, were observed at 3 months of age in the mt-mice (Figure 3A, the second panel from the left). At 6 and 12 months of age these glomerular lesions had progressed, and some mt-mice exhibited renal failure with severe glomerulosclerosis (Figure 3A, the third and forth panels from the left) and inflammatory cell infiltration in the interstitium (data not shown). In addition, Van-Gieson staining revealed interstitial fibrosis (Figure S3A). Consistent with these observations, tissue fibrosis markers such as *crlf*, *TGF-β*, and *CTGF* were significantly elevated in the kidneys of the mt-mice compared with those of ht-mice (Figure S3B). We further analyzed the ultrastructure of the podocyte foot processes and the filtration glomeruli barrier by electron microscopy; the podocyte foot processes of mt-mice at 4 months old were remarkably flattened and fused compared with the well-shaped foot processes of the wt-mice (Figure 3B).

**Figure 4:**
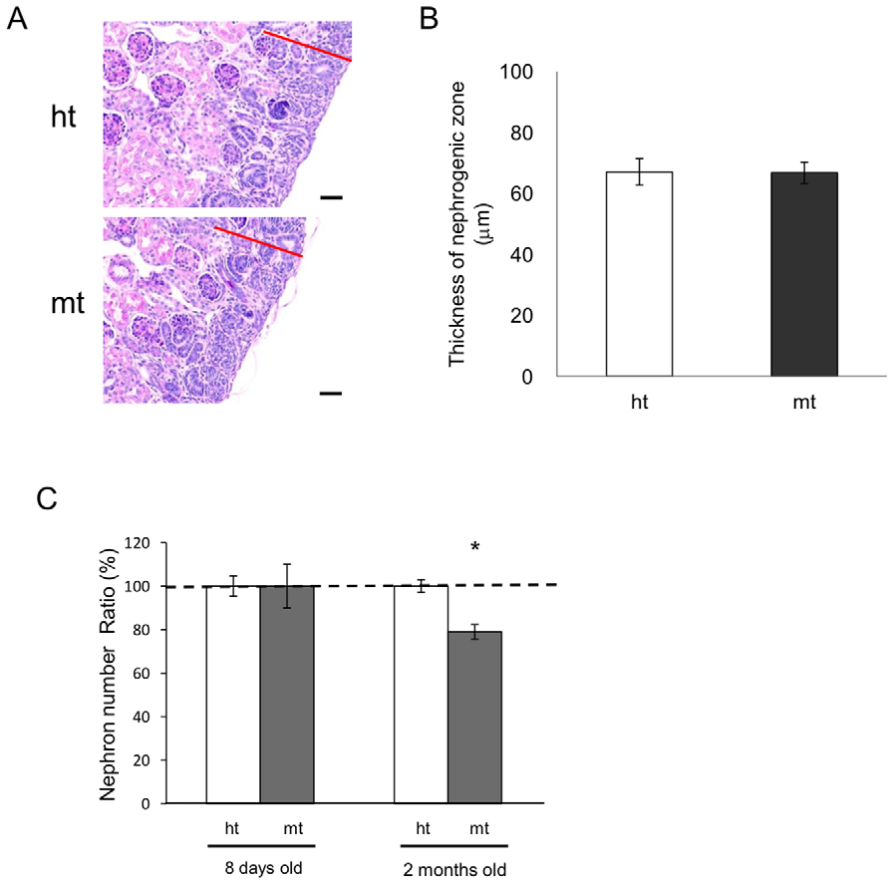
Nephrogenic zone formation and nephron numbers in kidneys. (A) PAS-stained kidney sections of ht (upper panel) and mt (lower panel) mice at 3 days after birth. Red bars indicate the nephrogenic zone. (B) Nephrogenic zone thickness compared between ht (open bars, n = 4) and mt (closed bars, n = 3) mice at 3 days after birth. (C) Relative nephron numbers compared between ht (open bars) and mt (closed bars) mice at 8 days (n = 10 ht, 9 mt) and 2 months (n = 6 ht, 6 mt) of age. Nephron number per mm^2^ in the ht renal cortex at each age was designated as 100%. *P<0.05 (Mann-Whitney U-test).

Since glomerular lesions were observed shortly after birth, we examined whether nephron development was impaired. The size of the nephrogenic zone, where nephrons develop, and the number of nephrons in the cortex at 3 and 8 days after birth, respectively, were comparable between mt- and ht-mice, while the number of nephrons was significantly reduced in the 2-month-old mt-mice (Figure 4). These observations suggest that nephron development was normal in the mt-mice, but nephron maintenance was impaired.

### Lectin staining of kidneys

**Figure 5:**
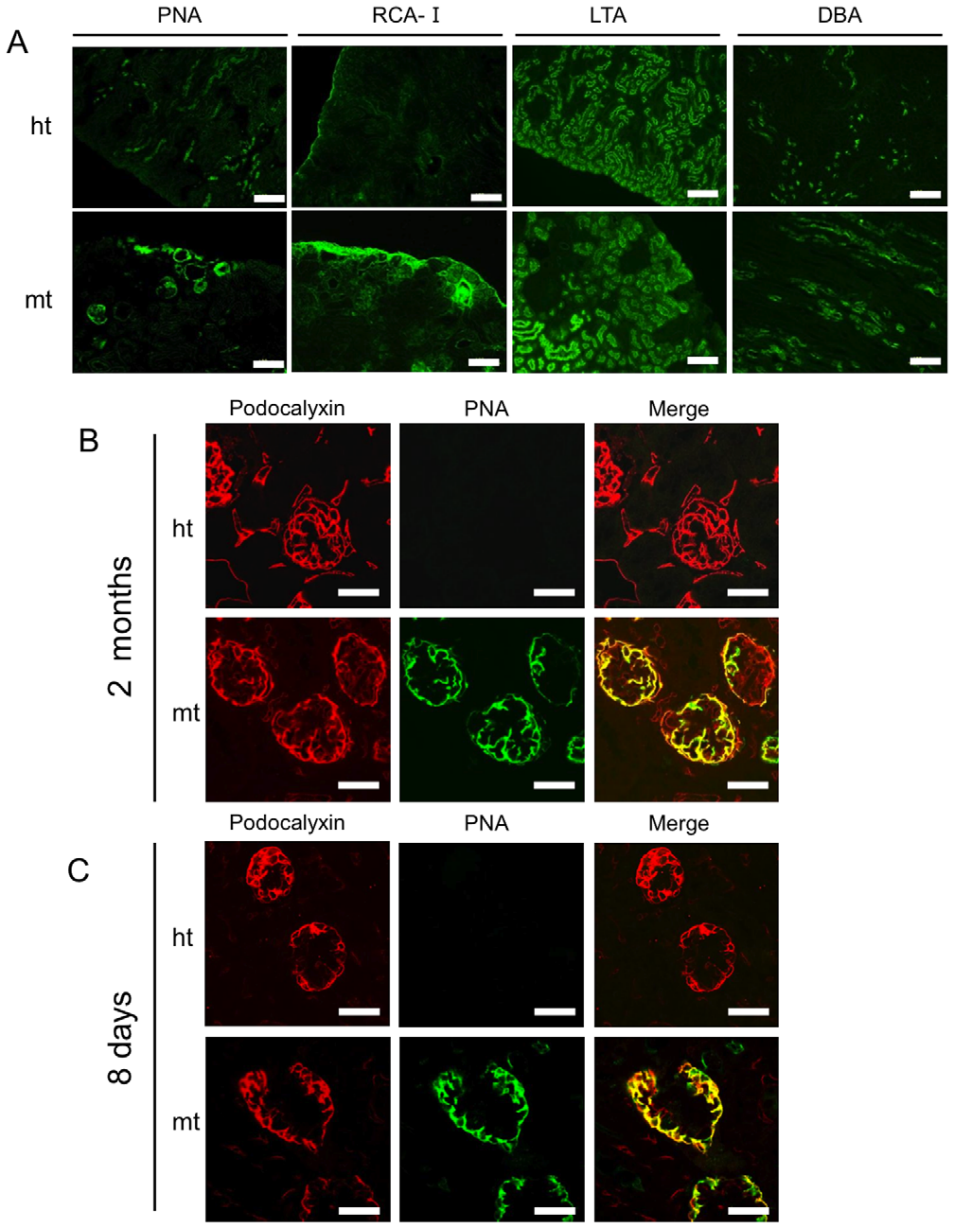
Lectin staining and glycan analysis of podocalyxin (PC) in kidneys. (A) Kidney sections stained with PNA, RCA-I, LTA, and DBA lectins (from left panels to right panels, respectively) in 3-month-old ht (upper panels) and mt (lower panels) mice. Scale bars: 120 µm. (B) Confocal laser scanning microscopic analysis of kidneys double-stained for PC and PNA, from 2-month-old ht (upper panels) and mt (lower panels) mice. Sections were stained with an anti-PC antibody (left panels; red), PNA (middle panels; green), and both (right panels; merge). Scale bars: 50 µm. (C) Confocal laser scanning microscopic analysis of kidneys double-stained for PC and PNA, from ht (upper panels) and mt (lower panels) mice at 8 days after birth. Sections were stained with the anti-PC antibody (left panels), PNA (middle panels; green), and both (right panels; merge). Scale bars: 50 µm.

To investigate the cell-surface glycan structures in the mt-mouse kidneys, we stained sections with several lectins, including PNA for Galβ1-3GalNAc, RCA-I for Galβ1-4GlcAc, LTA for L-Fucose, and DBA for GalNAcα1-3GalNAc (Figure 5A). In the mt-mouse kidneys, PNA-positive signals were aberrantly detected in glomeruli, and the RCA-I signals were more intense than in ht-mouse kidneys. High-magnification confocal microscopy revealed that PNA colocalized well with the epithelial cell marker podocalyxin (PC) in the mt-mouse glomeruli, while PNA was not observed in the ht-mouse glomeruli (Figure 5B). In contrast, PNA did not colocalize with the endothelial cell marker LEL in mt-mice; this indicates that PNA localized to glomeruli epithelial cells (Figure S4). PNA was observed in even immature glomeruli in 8-day-old mt-mice, but not in ht-mice (Figure 5C). These abnormal PNA and RCA-I signals suggested that glycoproteins and glycolipids in the mt-mouse kidneys, particularly in glomerular epithelial cells, were hyposialylated, since PNA and RCA-I recognize asialo glycans. On the other hand, the staining patterns of LTA and DBA, which recognize glycans with no relation to sialic acid, were comparable between the mt- and ht-mice, suggesting that the glycan structures recognized by LTA and DBA were not affected in the mt-mice.

### Hyposialylation of a major podocyte sialoprotein, podocalyxin (PC) in the mt-mice

**Figure 6:**
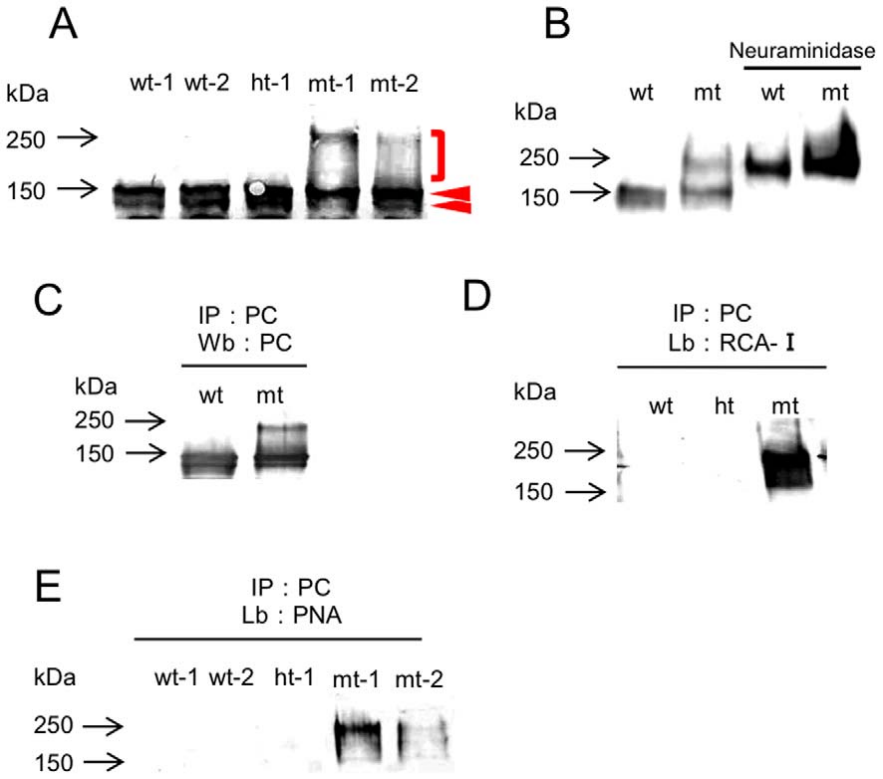
Western blot and lectin blot PC analysis. (A) Western blotting analysis of kidney lysates, using an anti-PC antibody. PC bands in wt and ht mice were about 140–150 kDa (arrowheads), while those in mt mice showed a shift to higher molecular weights (bracketed). (B) Neuraminidase treatment shifted the PC bands in wt mice to higher molecular weights similar to those seen in untreated mt mice. (C to E) Glycan analysis of PC by lectin blotting. After immunoprecipitation with an anti-PC antibody, PC bands were detected by PC (C), RCA-I (D), and PNA (E).

Western blotting analysis of several renal proteins was performed using whole kidney lysates. There was no difference in the band intensity or mobility of NHERF2, ezrin, nephrin, podocin, podoplanin, or NEPH1 between the ht- and mt-mice (Figure S5). However, while 140–150 kDa bands of PC were detected in lysates from both the control and mt-mice, the lysates from mt-mice also produced higher molecular-weight 250-kDa smear bands by appearance (Figure 6A). Since sialoprotein hyposialylation gives rise to smear bands with higher molecular weights than expected, we treated the PC with neuraminidase (sialidase). The PC bands in neuraminidase-treated lysates from wt-mice shifted to about 250 kDa, much like the PC bands in untreated lysates from mt-mice (Figure 6B). We further investigated the PC glycan chains in the mt-mice by lectin blotting. The 250-kDa smear band could be detected in mt-mice lysates after immunoprecipitation with the anti-PC antibody (Figure 6C), and was detected only in these samples by the lectins RCA-I and PNA, which recognize asialo glycans (Figure 6D, E). These results collectively indicate that the PC in mt-mouse kidneys was highly hyposialylated.

### Suppression of the renal disorders in mt-mice by Neu5Ac administration

If the hyposialylation of PC and other sialoproteins is a major cause of the renal disorders in mt-mice, administering Neu5Ac from early developmental stages onward can suppress disease development. Pregnant ht-mice were given Neu5Ac in their drinking water, and their mt-pups were given Neu5A-containing water according to the experimental schedule (Figure S2). At 2 months old, mt-mice and ht-mice, untreated or Neu5Ac-treated, were sacrificed for histological and urinary analysis.

**Figure 7:**
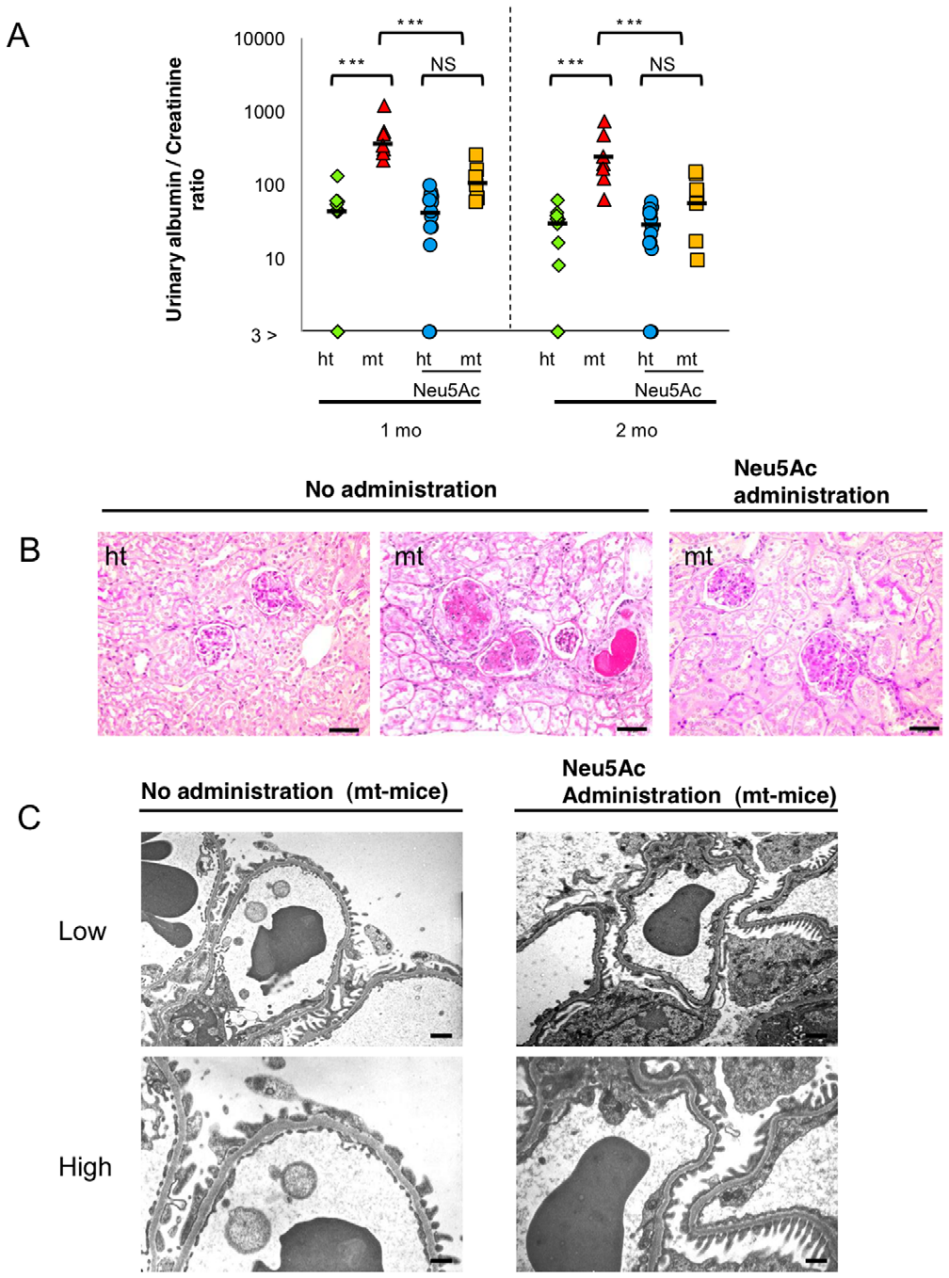
Urinary and histological analysis of mt mice treated with Neu5Ac. (A) Urinary albumin/creatinine ratios in untreated ht (green diamonds, n = 10) and mt (red triangles, n = 7) mice, and in Neu5Ac-treated ht (blue circles, n = 13) and mt (yellow squares, n = 8) mice at 1 and 2 months of age. ***P<0.001 (Two-way ANOVA). (B) PAS-stained kidney sections of untreated ht (left panel) and mt (middle panel) mice, and of Neu5Ac-treated mt (right panel) mice at 2 months old. Scale bars: 20 µm. (C) Electron microscopy of the glomeruli of mt mice at 2 months old that were untreated (left panels) or treated (right panels) with Neu5Ac. Low (upper panels, scale bars: 1 µm) and high magnification pictures (lower panels, scale bars: 500 nm).

The urinary albumin/creatinine ratio of the Neu5Ac-treated mt-mice was significantly reduced compared with untreated mt-mice, and was not significantly different from that of the Neu5Ac-treated ht-mice (Figure 7A). PAS-stained kidney sections of untreated and Neu5Ac-treated ht-mice appeared normal (Figure 7B left panel, and data not shown). Although the kidneys of untreated mt-mice were impaired, as described in Figure 3A (Figure 7B, middle panel), those of the Neu5Ac-treated mt-mice were much less affected. Enlarged glomeruli and dilatation of Bowman's spaces were rarely observed, and the mesangial matrix deposits were milder in the kidneys of the Neu5Ac-treated, compared with the untreated, mt-mice (Figure 7B right panel). Ultrastructural analysis showed that most of the podocyte foot processes in the Neu5Ac-treated mt-mice were well formed, and flattened and fused foot processes were observed less often than in untreated mt-mice (Figure 7C).

**Figure 8:**
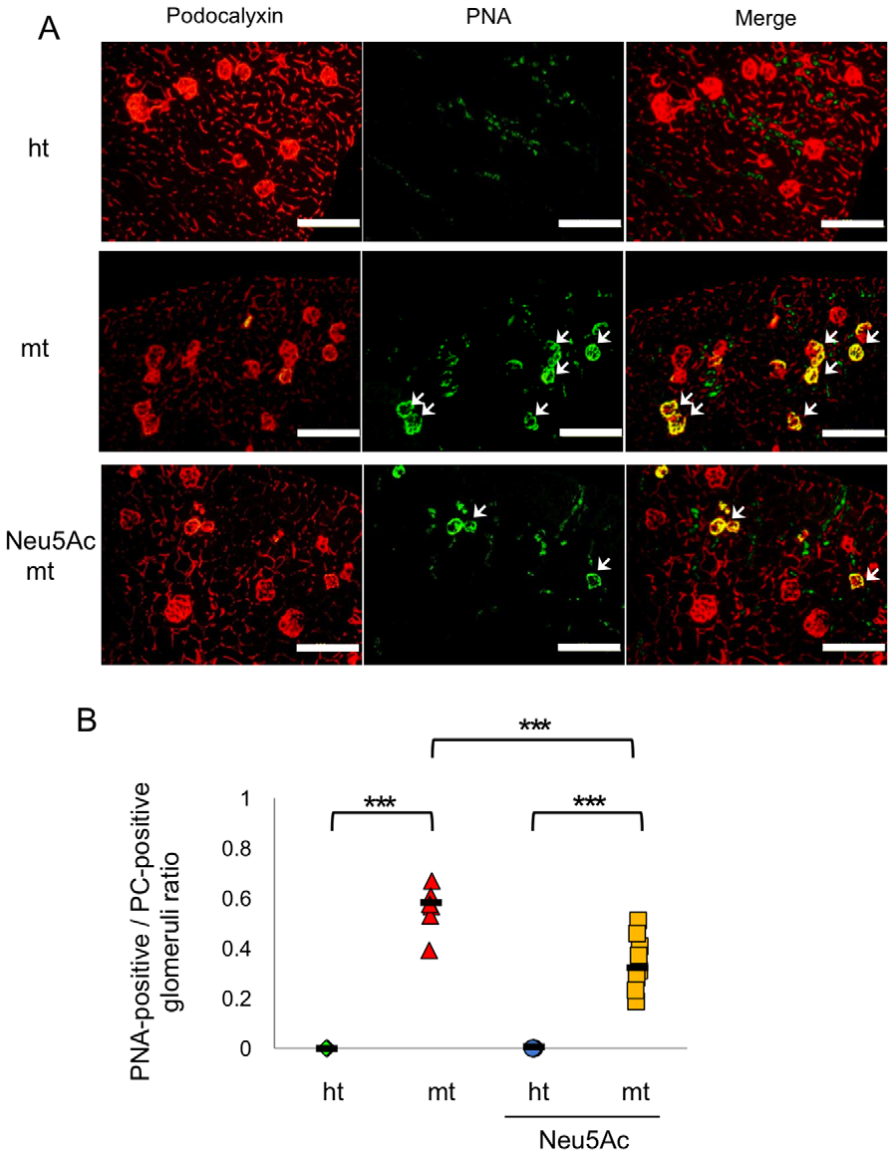
Reduced PNA-positive glomeruli ratios in Neu5Ac-treated mt mice. (A) Kidneys double-stained for PC and PNA, from untreated ht (upper panels) and mt (middle panels) mice, and Neu5Ac-treated mt mice (bottom panels). Sections were stained with an anti-PC antibody (left panels; red), PNA (middle panels; green), and both (right panels; merge). Scale bars: 200 µm. (B) Ratio of PNA-positive to PC-positive glomeruli in the kidneys of 2-month-old untreated ht (green diamonds, n = 4) and mt (red triangles, n = 6) mice, and of Neu5Ac-treated ht (blue circles, n = 5) and mt (yellow squares, n = 8) mice. ***P<0.001 (Two-way ANOVA).

Since PC can be used as a marker for glomerular epithelial cells, we double-stained kidney sections with an anti-PC antibody and PNA to estimate the ratio of PNA-positive to total glomeruli (Figure 8). While PNA-positive glomeruli were not detected in ht-mice, a large number of glomeruli were positive for PNA in the mt-mice (0% vs 56%) (Figure 8A, B). Neu5Ac treatment significantly reduced the proportion of PNA-positive glomeruli in the mt-mice (35%), indicating that Neu5Ac administration partially recovered the sialylation of PC and/or other glomerular glycoproteins and glycolipids.

## Discussion

GNE is a dual-functioning enzyme, and various *GNE* gene point mutations have been identified in DMRV patients. To elucidate the pathological mechanisms of GNE point mutations and their effect on sialic acid biosynthesis, we generated mice with a GNE V572L point mutation found in Japanese DMRV patients [3]. We showed that the GNE V572L mt-mice had a shorter lifespan than wt- and ht-mice, and that they developed renal disorders with massive proteinuria shortly after birth. However, these mice did not exhibit apparent myopathies and motor dysfunction seen in DMRV (Figure S1B, S1C), and no renal disorder has been reported in DMRV patients. It is still not clear why the same V572L GNE point mutation caused different diseases in mice than those seen in humans. One possibility is that hyposialylation causes the dysfunction of different tissues and cells in mice than in humans. To know such differences, it will be very informative to examine the sialylation status of glomeruli, especially the PC sialylation status, in human DMRV patients. Another possibility is that the molecular Neu5Ac and Neu5Gc sialic acid species might function differently in humans and mice, since humans, but not mice, are genetically defective in synthesizing Neu5Gc, a common mammalian sialic acid [34].

Furthermore, the phenotype of our mice was different than that of the two previously reported GNE point-mutant mice, a transgenic mouse carrying a human D176V point-mutant *GNE* gene in a mouse *GNE* gene-knockout background [20], [21], and a knockin mouse carrying a GNE M712T point mutation [18]. The D176V GNE point-mutant mice exhibit myopathic features, but not renal disorders, while the M712 T point-mutant mice display severe renal hematuria and neonatal lethality, but not myopathy. The genetic situation of the D176V point-mutant mouse is different from the other two GNE mutant mice and from human DMRV patients, because the introduced human D176V point-mutant *GNE* gene is over-expressed. The M712T point-mutant mice suffer from far more severe renal disorders than those seen in our mice. It is not certain why the renal disorders differ between the two GNE-mutant mice. Although the V572L and M712T mutations are both located in the GNE kinase domain, they might affect different GNE functions. The V572L mutation may interfere with kinase domain dimerization, while the M712T mutation may change the GNE structure to affect in ATP catalysis, carbohydrate binding, and phosphoryl transfer [35]. These differences might affect the degree of hyposialylation of glycoproteins and glycolipids including PC.

Our mt-mice displayed glomerular defects with massive proteinuria shortly after birth, and had abnormally flattened and diffused podocyte foot processes. Their lifespan was significantly shortened by renal failure. These features resemble CNS, a very rare type of nephrotic syndrome. It is identified primarily in families of Finnish origin. Children born with the Finnish type of CNS die within a few months after birth due to massive proteinuria caused by impaired podocyte function [17], [36], [37]. The causative genes, *NPHS1*, which encodes nephrin and *CD2-accosiated protein*, which encodes CD2AP, have been identified in humans and mice [36], [38]–[40]. These genes are expressed in glomerular epithelial cells in the kidney. Nephrin-deficient pups die with severe proteinuria immediately after birth. The kidneys of nephrin-deficient mice show fibrotic and hypercellular glomeruli, enlarged Bowman's spaces, dilated tubules, effaced podocyte foot processes, and the absence of slit-diaphragms, all characteristic features seen in human CNS. In heterozygous mice, approximately one third of the foot processes are fused, and nephrin mRNA levels are reduced [17], [22], [40]. On the other hand, CD2AP-deficient pups show proteinuria from two weeks of age onward, and most die of renal failure at six to seven weeks of age. They exhibit defective podocyte foot processes, accompanied by mesangial cell hyperplasia and extracellular matrix deposits similar to that found in nephrin-deficient mice. CD2AP associates with nephrin to form components of the slit diaphragms. Furthermore, heterozygous CD2AP mice also show proteinuria and glomerulosclerosis-like damage at nine months of age [17], [22], [38], [39]. Therefore, the histological and pathological characteristics of our mt-mice resembled those of nephrin-deficient and CD2AP-deficient mice. However, our mt-mice survived longer than these CNS model mice, and our ht-mice did not show adverse effects in the kidneys, suggesting that the GNE V572L point mutation had a milder effect on renal pathologies than that seen in the CNS model mice.

The formation of the nephrogenic zone and the number of nephrons in the renal cortex were comparable between neonatal mt-mice and ht-mice, suggesting that nephron development was not impaired and that nephronophthisis did not occur (Figure 4). On the other hand, by two months of age, the mt-mice had significantly fewer nephrons than did ht-mice, suggesting that nephron maintenance in these mice was affected by the glomerular damage with massive albuminuria. The remarkably enlarged glomeruli in the mt-mice might be caused by compensatory effects of the reduced number of nephrons.

The PNA and RCA-I lectin-staining patterns in the kidneys of the mt-mice were considerably different from those in the ht-mice (Figure 5); PNA-positive signals were detected in the glomeruli, and the RCA-I signals were more intense in the mt-mouse kidneys. PNA-positive glomeruli are also observed in human glomerular disease, due to increased endogenous glomerular sialidase, and in diabetic nephropathy due to disturbed glycan turnover [41], [42]. Since galactose-containing epitopes, Galβ1-3GalNAc and Galβ1-4GlcAc, which are recognized by PNA and RCA-I, respectively, are usually highly sialylated, these lectins cannot recognize these sialylated epitopes in ht-mice. Therefore, our results suggest that the GNE V572L mutation causes glycoproteins and glycolipids in the glomeruli to be highly hyposialylated. In western blot analyses of various glomerular epithelial proteins, only PC showed a mobility shift in the mt-mice. Neuraminidase treatment, immunoprecipitation/lectin blotting, and the colocalization of PC and PNA in the glomeruli confirmed that PC was highly hyposialylated in the mt-mouse kidney. PC hyposialylation is also observed in M712T mutant mice [18], suggesting that certain *GNE* point mutations cause PC hyposialylation. Since PC is a highly sialylated glycoprotein, its hyposialylation is easily detected by a mobility shift. We cannot rule out, however, the possibility that the sialylation of other glomerular epithelial proteins was slightly affected by the GNE mutation.

PC is a CD34-related sialomucin that is strongly expressed in podocytes. The negative charge of its highly sialylated extracellular domain makes PC important for maintaining the characteristic architecture of foot processes and the patency of slit-diaphragms [13], [14], [43]–[45]. PC-null mice die of anuric renal failure within twenty-four hours of birth [46]. They fail to form foot processes and slit-diaphragms, which impairs urine production. Interestingly, *CD34* and *endoglycan* mRNA expression in the kidneys was about two-fold higher in the mt-mice than in ht-mice, while the *PC* mRNA levels were comparable between the two (Figure S6). *CD34* mRNA levels are also increased in the kidneys of *PC-*null mice compared to wt-mice [46]. These results suggest that PC protein dysfunction due to hyposialylation may cause a compensatory increase in the related sialomucins CD34 and endoglycan. Takeda *et al.* [19] reported that PC hyposialylation or desialylation in rats, generated by treatment with reagents such as puromycin aminonucleoside, disrupts PC complexes containing PC, NHERF2 and ezrin, and their interactions with the actin cytoskeleton, resulting in a loss of foot processes similar to that seen in human nephrotic syndrome. Therefore, hyposialylated PC could be a cause of the nephrotic-like syndrome in mt-mice.

To clarify whether hyposialylation is the cause of the nephrotic-like syndrome in mt-mice, we conducted a therapeutic study by administering sialic acid metabolites to mt-mice from embryonic stages through two months of age. Since ManNAc phosphorylation is affected by the GNE V572L point mutation in the sialic acid biosynthesis pathway [35], Neu5Ac, a final product in the pathway, rather than ManNAc, was administrated in the therapeutic study. Neu5Ac treatment markedly suppressed the albuminuria and renal disorders, including fused podocyte foot processes, dilatation of Bowman's space and glomerulosclerosis, in the mt-mice. The proportion of PNA-positive glomeruli was reduced, indicating that PC sialylation was recovered. Therefore, starting Neu5Ac treatment prenatally prevented mt-mice from developing the nephrotic-like syndrome. These results suggest that the hyposialylation of renal proteins, including PC, causes the nephrotic-like syndrome in the mt-mice. Similar therapeutic studies using sialic acid metabolites have been demonstrated in GNE D176V and GNE M712T-mice, with the amelioration of myopathies and renal hematuria, respectively [18], [21].

In conclusion, the GNE V572L mutation caused the hyposialylation of renal glycoproteins such as PC, resulting in glomerular filtration barrier failure and nephrotic syndrome-like phenotypes. Our mt-mice are suitable for long-term therapeutic trials and the pathological analysis of nephrotic-like syndromes. We consider the negatively charged monosaccharide Neu5Ac to be a promising therapeutic tool for some nephrotic syndromes; candidate disorders include focal and segmental glomerulonephritis [42], [47], membranous glomerulopathy [42], and other unexplained nephrotic syndromes, congenital or otherwise. However, no renal diseases caused by hyposialylation have been identified to date, and renal abnormality has not been reported in DMRV patients. Several issues still need to be addressed, such as the pathomechanisms of the massive proteinuria caused by the hyposialylated glomerular glycoproteins and glycolipids, and the therapeutic effect of other sialic acid metabolites, in our mt-mice.

## Supporting Information

Figure S1 **PCR genotyping, skeletal muscle histology, and rota-rod tests.** (**A**) Mice were genotyped by PCR using allele-specific locked nucleic acid (LNA™)-containing primers. A wild-type band (153 bp) of the *GNE* gene was detected by wild-type allele-specific primers (**left panel**), and a mutant band (153 bp) was detected by mutant allele-specific primers (**right panel**). (**B**) Modified Gomori's trichrome-stained sections of quadriceps femoris muscles of 6-month-old ht (**left panel**) and mt (**right panel**) mice. (**C**) Motor coordination and learning was assessed by an accelerating (5–40 rpm) rota-rod paradigm (wt, green circles; ht, blue triangles; mt, red squares).
(TIF)

Figure S2 **Scheme of the therapeutic Neu5Ac experiment.** Pregnant ht mice were not treated or were treated with 1 g/kg/day of Neu5Ac in the drinking water, from mating through the nursing period. After weaning, pups were not treated or were treated with Neu5Ac (0.2 g/kg/day) in the drinking water until they were 2 months old. The mice were sacrificed at 2 months of age and were analyzed by histological and biochemical methods. Urine was collected at 1 and 2 months of age.
(TIF)

Figure S3 **Kidney fibrosis.** (**A**) Van-Gieson-stained kidney sections of ht (**upper panels**) and mt (**lower panels**) mice at 3 months (**left panels**) and 12 months (**right panels**) of age. (**B**) Quantitative RT-PCR analysis of the expression levels of several genes implicated in renal fibrosis in 3-month-old ht (n = 6) and mt (n = 5) mice. The expression level of each gene was normalized to that of the *GAPDH* gene. Results are shown as means ± SD. *P<0.05, **P<0.01 (student's *t*-test).
(TIF)

Figure S4 **Lectin staining analysis of glomerular endothelial cells.** Confocal laser scanning microscopic analysis of double-staining for LEL, a marker for endothelial cells, and PNA in the kidneys of 3-month-old ht (**upper panels**) and mt (**lower panels**) mice. Sections were stained with LEL (**left panels; green**), PNA (**middle panels; red**), and both (**right panels; merge**). Scale bars: 25 µm.
(TIF)

Figure S5 **Western blot analysis of podocalyxin-related proteins expressed in podocytes.** Protein bands were detected at the expected molecular sizes and similar intensities in the ht and mt mice: NHERF2 (40 kDa), ezrin (80 kDa), nephrin (100 kDa), podocin (42 kDa), podoplanin (38 kDa), and NEPH1 (65 kDa).
(TIF)

Figure S6 **Expression levels of CD34 family genes in the kidneys.** Expression levels of CD34 family genes were analyzed in the kidneys of 6-month-old ht (open bars, n = 5–6) and mt (closed bars, n = 5–6) mice by quantitative RT-PCR. Expression levels of each gene were normalized to those of the *GAPDH* gene. Results are shown as means ± SD. *P<0.05, **P<0.01 (student's *t*-test).
(TIF)

Table S1 **List of primers used for QRT-PCR.**
(TIF)
