# Supplementary figures and images for: Glycoprotein Hyposialylation Gives Rise to a Nephrotic-Like Syndrome That Is Prevented by Sialic Acid Administration in GNE V572L Point-Mutant Mice
Source: PLoS One. 2012 Jan 13;7(1):e29873. doi: 10.1371/journal.pone.0029873 (PMC3258264; doi:10.1371/journal.pone.0029873)

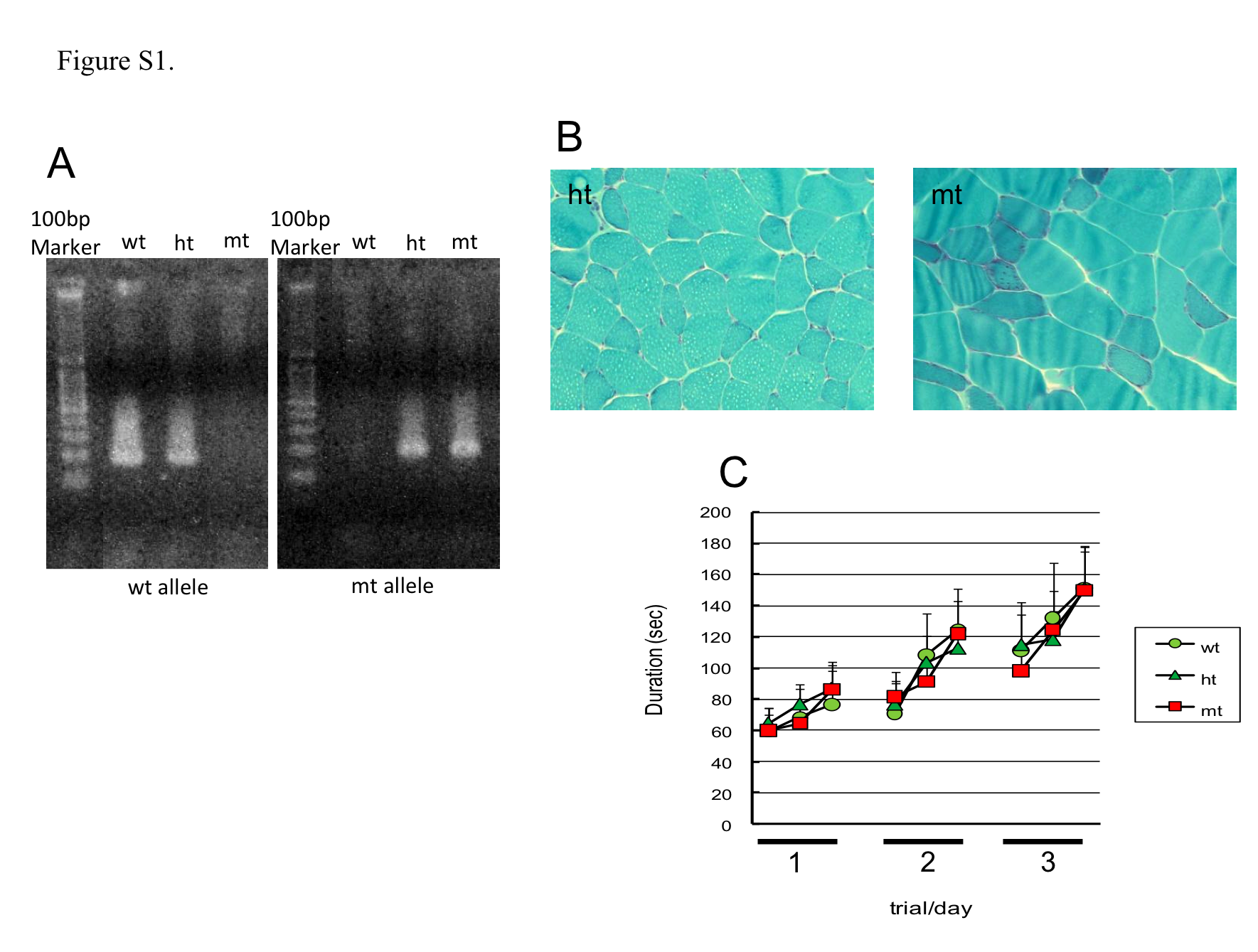

Supplement: Figure S1 — PCR genotyping, skeletal muscle histology, and rota-rod tests. (A) Mice were genotyped by PCR using allele-specific locked nucleic acid (LNA™)-containing primers. A wild-type band (153 bp) of the GNE gene was detected by wild-type allele-specific primers (left panel), and a mutant band (153 bp) was detected by mutant allele-specific primers (right panel). (B) Modified Gomori's trichrome-stained sections of quadriceps femoris muscles of 6-month-old ht (left panel) and mt (right panel) mice. (C) Motor coordination and learning was assessed by an accelerating (5–40 rpm) rota-rod paradigm (wt, green circles; ht, blue triangles; mt, red squares). (TIF) [file pone.0029873.s001.tif]

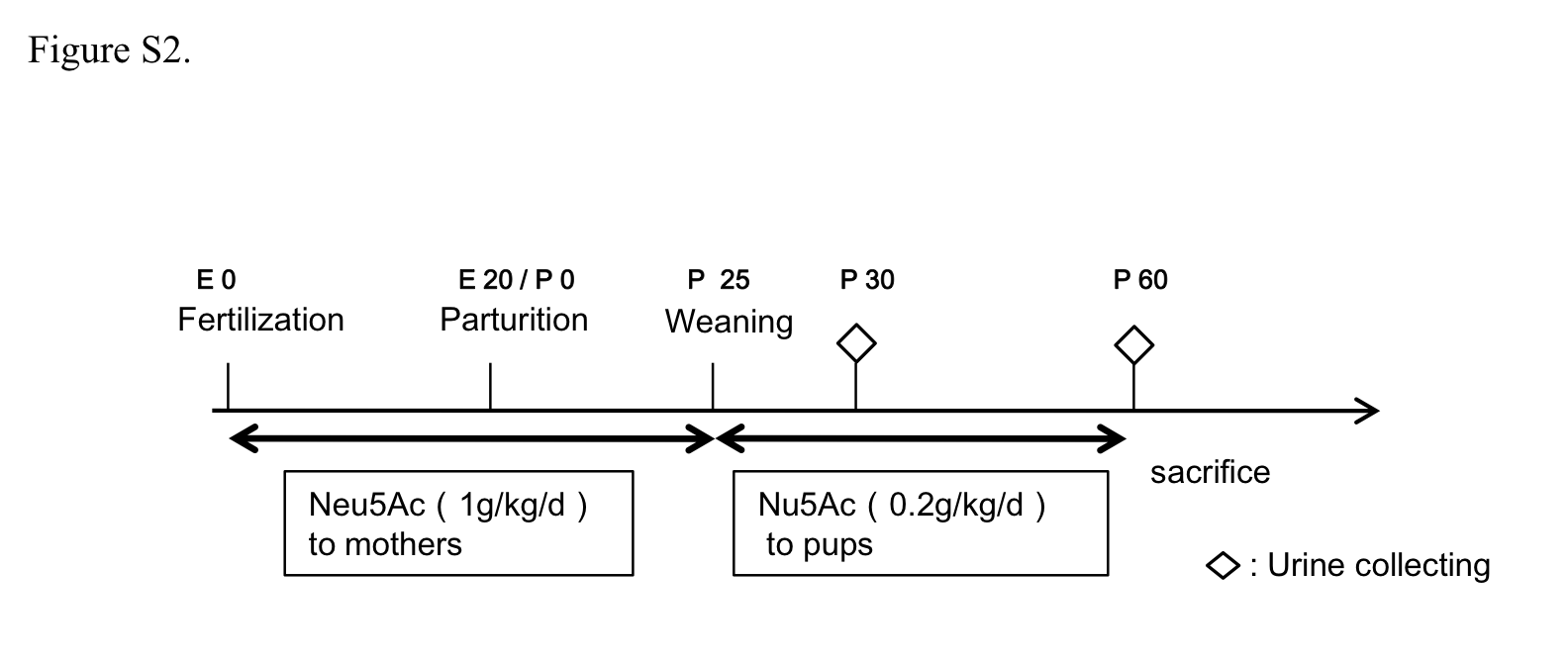

Supplement: Figure S2 — Scheme of the therapeutic Neu5Ac experiment. Pregnant ht mice were not treated or were treated with 1 g/kg/day of Neu5Ac in the drinking water, from mating through the nursing period. After weaning, pups were not treated or were treated with Neu5Ac (0.2 g/kg/day) in the drinking water until they were 2 months old. The mice were sacrificed at 2 months of age and were analyzed by histological and biochemical methods. Urine was collected at 1 and 2 months of age. (TIF) [file pone.0029873.s002.tif]

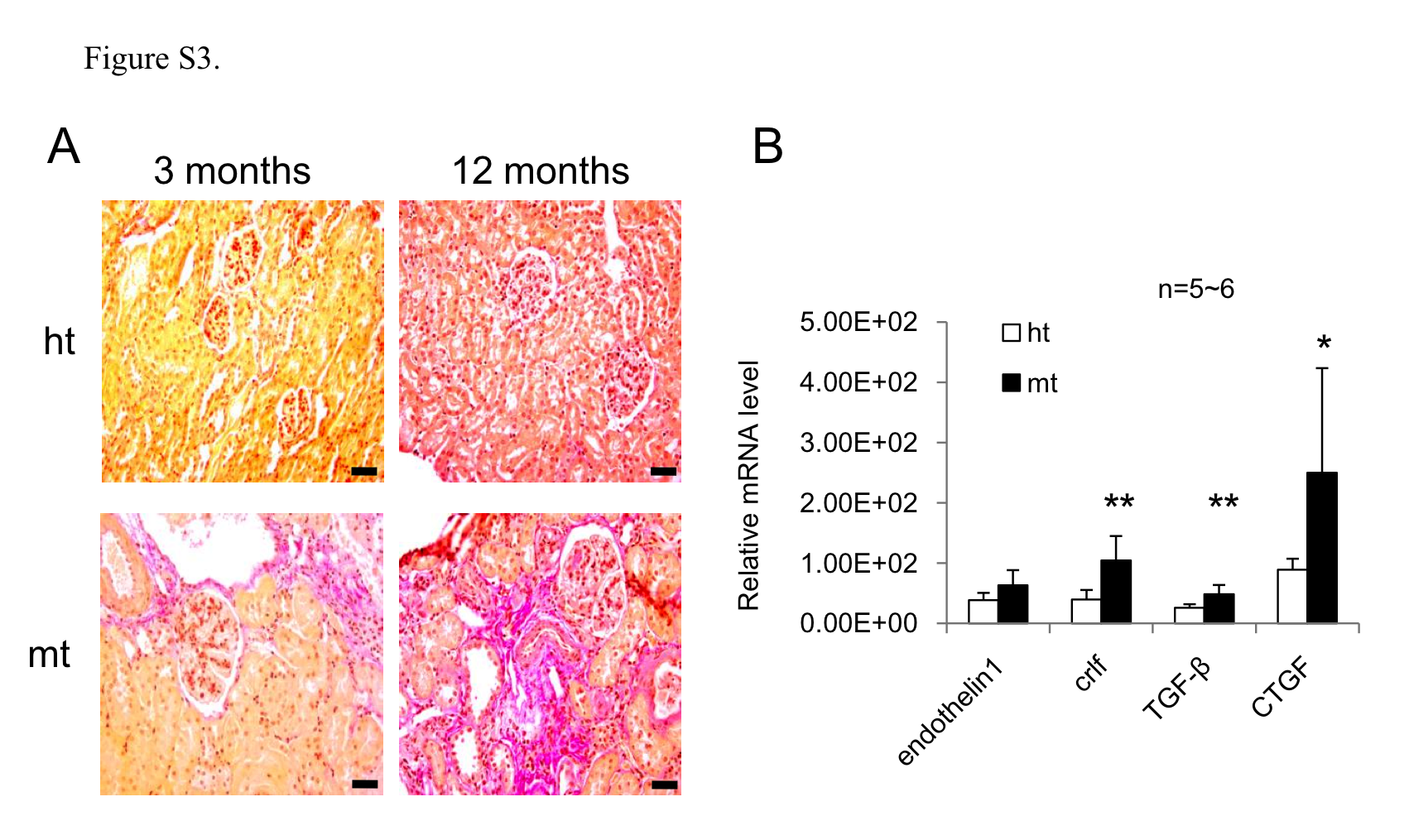

Supplement: Figure S3 — Kidney fibrosis. (A) Van-Gieson-stained kidney sections of ht (upper panels) and mt (lower panels) mice at 3 months (left panels) and 12 months (right panels) of age. (B) Quantitative RT-PCR analysis of the expression levels of several genes implicated in renal fibrosis in 3-month-old ht (n = 6) and mt (n = 5) mice. The expression level of each gene was normalized to that of the GAPDH gene. Results are shown as means ± SD. *P<0.05, **P<0.01 (student's t-test). (TIF) [file pone.0029873.s003.tif]

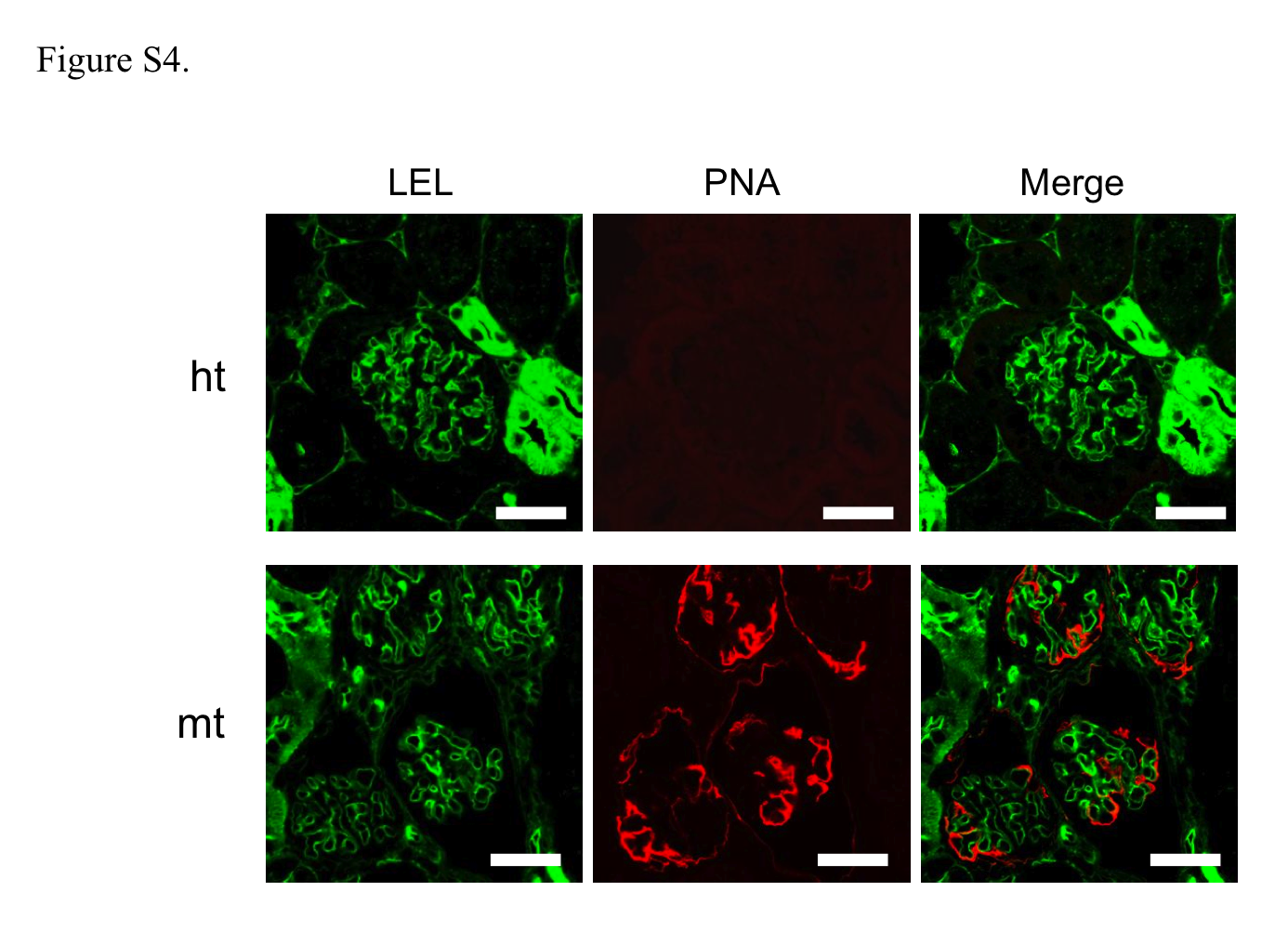

Supplement: Figure S4 — Lectin staining analysis of glomerular endothelial cells. Confocal laser scanning microscopic analysis of double-staining for LEL, a marker for endothelial cells, and PNA in the kidneys of 3-month-old ht (upper panels) and mt (lower panels) mice. Sections were stained with LEL (left panels; green), PNA (middle panels; red), and both (right panels; merge). Scale bars: 25 µm. (TIF) [file pone.0029873.s004.tif]

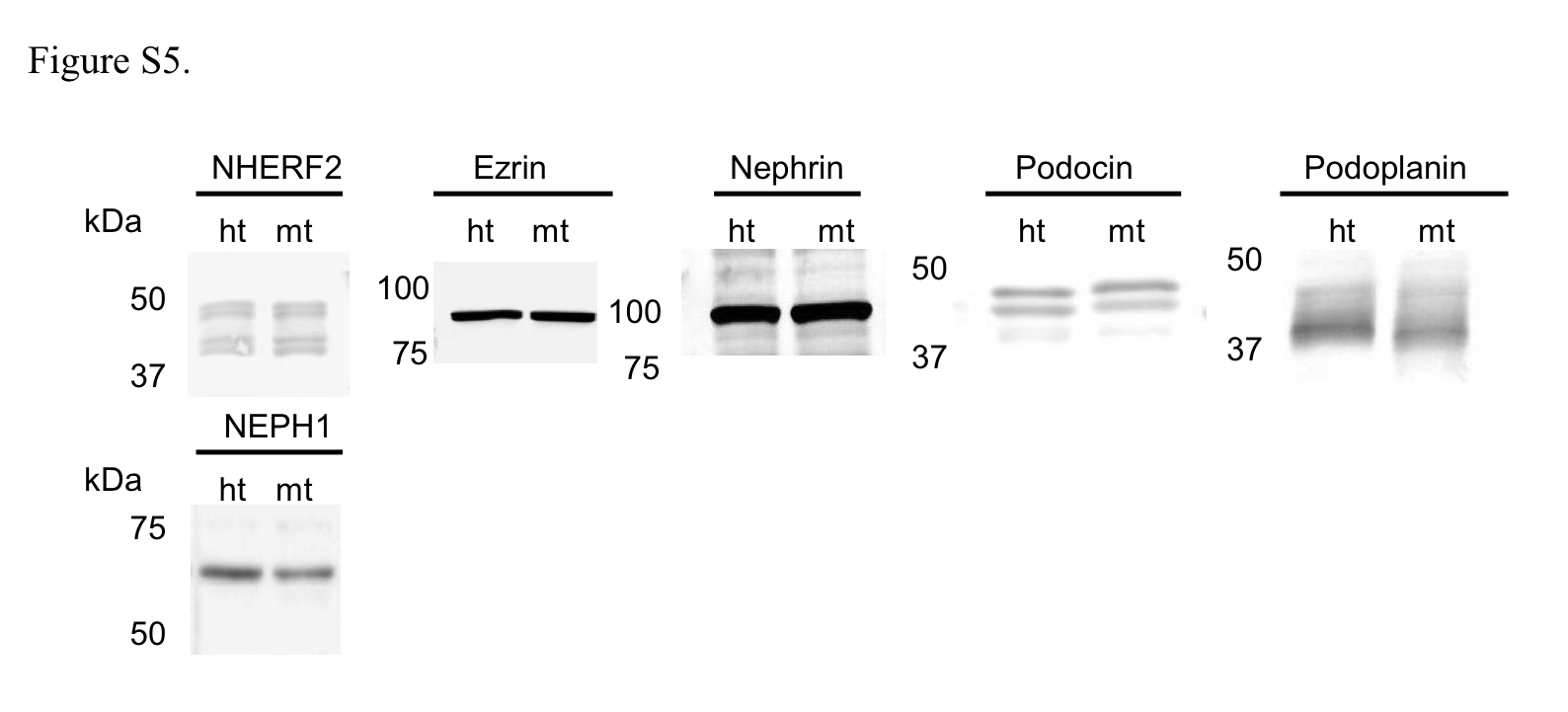

Supplement: Figure S5 — Western blot analysis of podocalyxin-related proteins expressed in podocytes. Protein bands were detected at the expected molecular sizes and similar intensities in the ht and mt mice: NHERF2 (40 kDa), ezrin (80 kDa), nephrin (100 kDa), podocin (42 kDa), podoplanin (38 kDa), and NEPH1 (65 kDa). (TIF) [file pone.0029873.s005.tif]

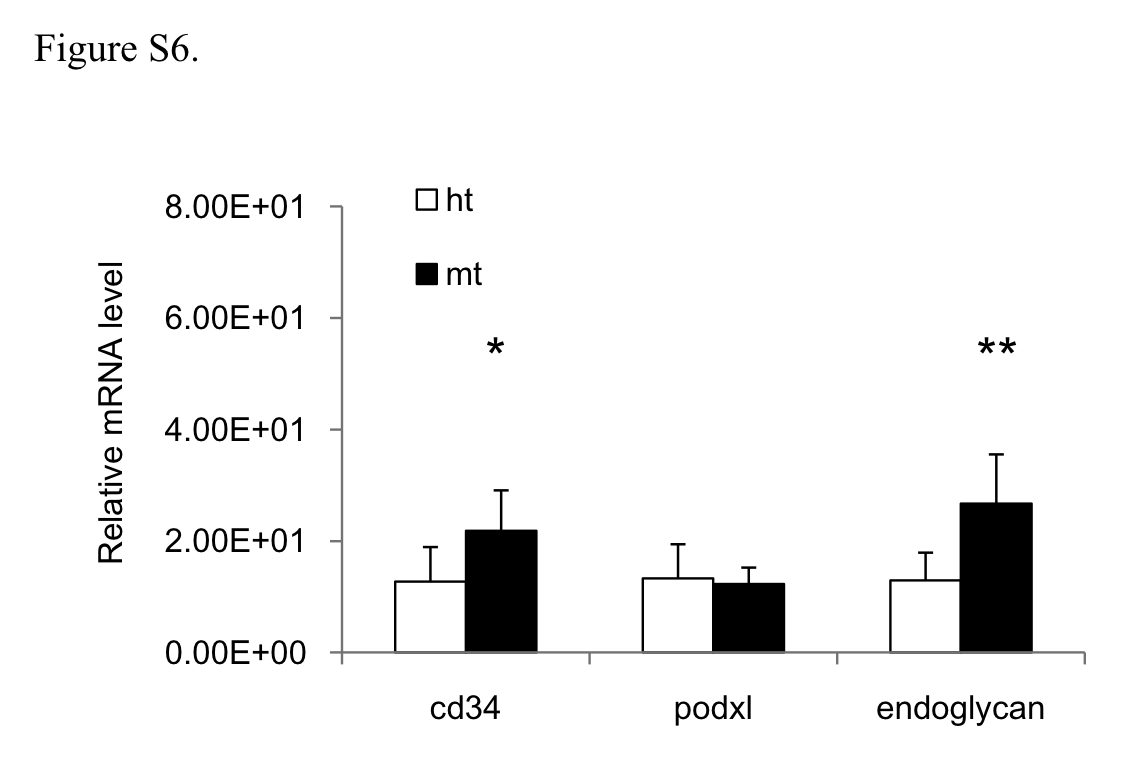

Supplement: Figure S6 — Expression levels of CD34 family genes in the kidneys. Expression levels of CD34 family genes were analyzed in the kidneys of 6-month-old ht (open bars, n = 5–6) and mt (closed bars, n = 5–6) mice by quantitative RT-PCR. Expression levels of each gene were normalized to those of the GAPDH gene. Results are shown as means ± SD. *P<0.05, **P<0.01 (student's t-test). (TIF) [file pone.0029873.s006.tif]

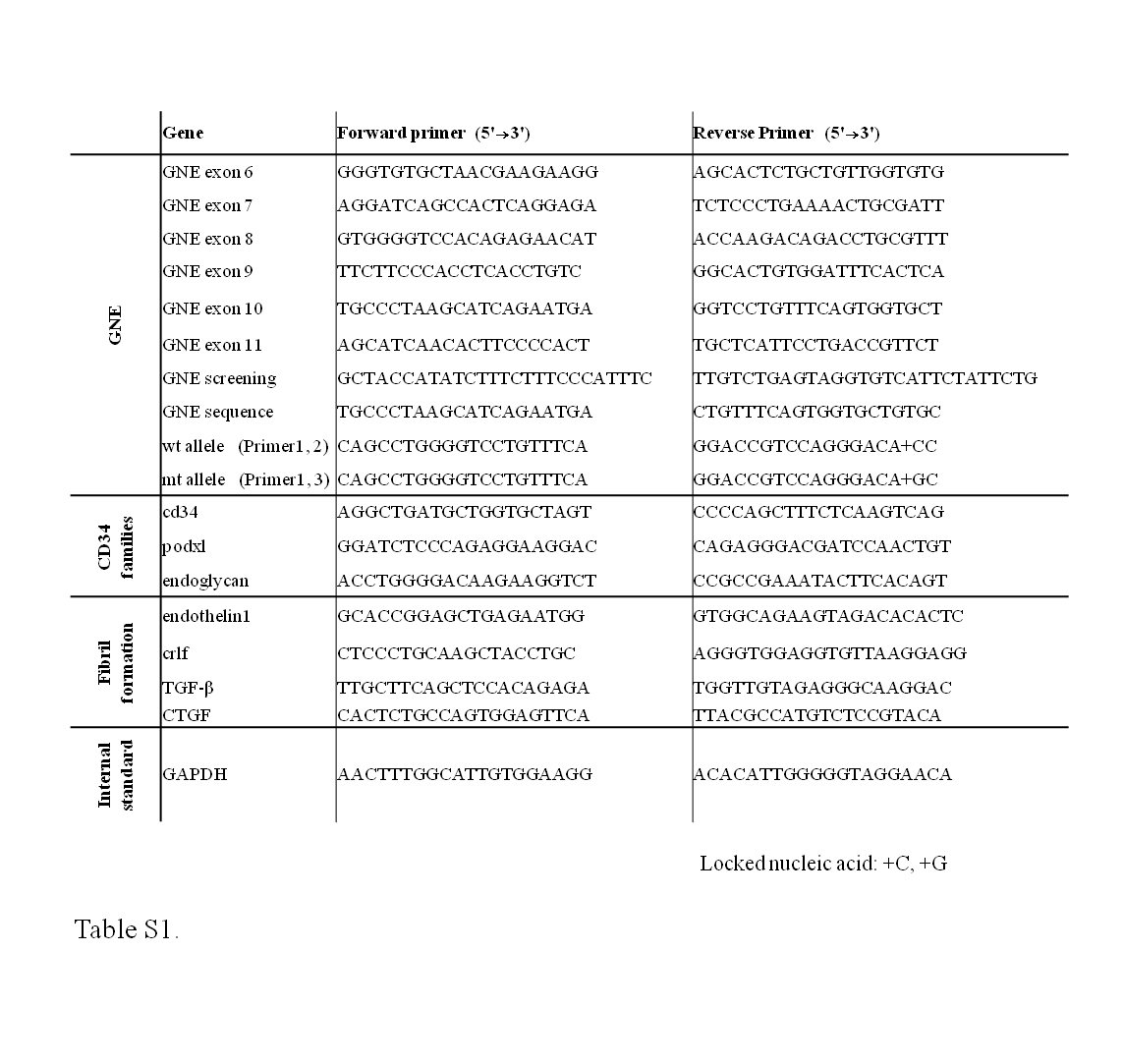

Supplement: Table S1 — List of primers used for QRT-PCR. (TIF) [file pone.0029873.s007.tif]
